# Robust Cubature Kalman Filter for Moving-Target Tracking with Missing Measurements

**DOI:** 10.3390/s24020392

**Published:** 2024-01-09

**Authors:** Samer Sahl, Enbin Song, Dunbiao Niu

**Affiliations:** 1 College of Mathematics, Sichuan University, Chengdu 610065, China; samersa7l@aun.edu.eg; 2 Department of Statistics, Assiut University, Assiut 71515, Egypt; 3 College of Electronic and Information Engineering, Tongji University, Shanghai 201804, China; dunbiaoniu_sc@163.com

**Keywords:** cubature Kalman filter, missing data, robust cubature Kalman filter

## Abstract

Handling the challenge of missing measurements in nonlinear systems is a difficult problem in various scientific and engineering fields. Missing measurements, which can arise from technical faults during observation, diffusion channel shrinking, or the loss of specific metrics, can bring many challenges when estimating the state of nonlinear systems. To tackle this issue, this paper proposes a technique that utilizes a robust cubature Kalman filter (RCKF) by integrating Huber’s M-estimation theory with the standard conventional cubature Kalman filter (CKF). Although a CKF is often used for solving nonlinear filtering problems, its effectiveness might be limited due to a lack of knowledge regarding the nonlinear model of the state and noise-related statistical information. In contrast, the RCKF demonstrates an ability to mitigate performance degradation and discretization issues related to track curves by leveraging covariance matrix predictions for state estimation and output control amidst dynamic disruption errors—even when noise statistics deviate from prior assumptions. The performance of extended Kalman filters (EKFs), unscented Kalman filters (UKFs), CKFs, and RCKFs was compared and evaluated using two numerical examples involving the Univariate Non-stationary Growth Model (UNGM) and bearing-only tracking (BOT). The numerical experiments demonstrated that the RCKF outperformed the EKF, EnKF, and CKF in effectively handling anomaly errors. Specifically, in the UNGM example, the RCKF achieved a significantly lower ARMSE (4.83) and ANCI (3.27)—similar outcomes were observed in the BOT example.

## 1. Introduction

Since its development in 1960 by Kalman [1], the Kalman filter has been widely utilized in ocean-atmosphere science to develop numerous nonlinear filters [2]. The EKF, UKF, EnKF, and CKF are commonly used variations of the Kalman filter [3]. The EKF linearizes nonlinear systems using the Jacobian matrix and first-order Taylor expansion, making it suitable for navigation, target tracking, data fusion, and state estimation [4]. However, the Jacobian matrix has limitations in achieving precise linearization with decreasing gradients [5]. The UKF, on the other hand, utilizes the unscented transform to avoid the need for computing the Jacobian matrix. However, it requires accurate prior knowledge of the system noise statistics, which can be challenging to describe correctly in dynamic environments, potentially leading to incomplete or divergent filtering solutions [6].

The EnKF belongs to the class of particle filters, where an ensemble of state estimates is selected to represent the initial probability distribution [7,8]. These estimates are propagated through the nonlinear system, approximating the probability density function of the true state [9]. However, for highly nonlinear applications requiring high precision and a finite ensemble size, the EnKF may not be optimal [10]. The CKF utilizes a third-degree cubature rule and offers advantages such as reduced parameters [11,12], improved stability, and accuracy compared to the UKF [13,14]. It is widely used to handle nonlinear problems [15], but applying the CKF to a nonlinear system requires knowledge of the mathematical model and noise statistics, which can be challenging to obtain in practical applications [16].

Over the last few years, considerable effort has been spent on developing the RCKF based on Huber’s idea of M-estimation and the traditional CKF. It can handle the problem of performance degradation, and the tracking curves are discretized whenever the data diverge from the previous noise statistics. Table 1 shows a comparison of relevant works for illustration.

Previous studies have shown that the RCKF algorithm provides significant improvements in tracking accuracy and stability in many applications, outperforming traditional methods. However, these studies failed to deal with the problem of missing measurements in nonlinear systems, which frequently occur in practical scenarios due to imprecise observations.

This paper suggests an RCKF technique based on Huber’s idea of M-estimation and the CKF for the estimation of the state of nonlinear systems with missing measurements. Missing measurements are often an inescapable occurrence in many practical scenarios due to the specific variables associated with erroneous observations. Interruptions in the technical aspects of observation, shrinkage occurrences in the diffusion channels, and erroneously lost measurements are some of the reasons for missing data. In addition, data inaccessibility is also a possibility [20,21]. To describe missing measurements using random variables, the Bernoulli distribution is more commonly used than the Markov chain [22]. We summarize the contributions and significance of this paper as follows:

1. The RCKF was developed by integrating Huber’s M-estimation theory with the standard CKF to effectively handle nonlinear systems, with missing measurements characterized using random variables following the Bernoulli distribution.
2. The RCKF exhibited superior performance compared to the EKF, EnKF, and CKF in terms of accuracy and reliability on two moving-target tracking models (UNGM and BOT) with missing measurements, indicating that the RCKF is the most effective approach for nonlinear systems with missing measurements.

This paper is organized as follows: Section 2 presents an analysis of a nonlinear system that is afflicted by missing measurements. Section 3 delves into the CKF and provides an in-depth discussion on Gaussian Bayesian filters. Section 4 proposes a novel approach to enhance the robustness of the CKF using robust estimation theory. In Section 5, a robust CKF is presented and applied in two numerical instances to compare its performance with existing filters. Finally, Section 6 concludes the paper.

## 2. Nonlinear System with Missing Measurements

The following equations formulate a nonlinear system with missing measurements [22]:

(1) $$\begin{array}{c} \lambda_{k}=g\left(\lambda_{k-1}\right)+\delta_{k-1}, \end{array}$$

(2) $$\begin{array}{c} \theta_{k}=\psi_{k}h\left(\lambda_{k}\right)+\xi_{k}, \end{array}$$

where *k* is the discrete time index; $\lambda_{k}\in R^{n}$ is the state vector; $\theta_{k}\in R^{m}$ is the measurement vector; $\delta_{k-1}\in R^{n}$ and $\xi_{k}\in R^{m}$ are process noise and measurement noise, respectively; g(·) and h(·) are the known nonlinear functions. Additionally, the nonlinear systems (1) and (2) are assumed to have the following properties:

1. The initial state follows a Gaussian distribution, i.e., $\lambda_{0}∼N\left({\bar{\lambda }}_{0},B_{0}\right)$ .
2. The noise sequences $\delta_{k-1}$ and $\xi_{k}$ are independent Gaussian sequences with zero means, and the covariance matrix of $\delta_{k-1}$ is denoted as $q_{k-1}$ , while the covariance matrix of $\xi_{k}$ is denoted as $r_{k}$ .

A Bernoulli distribution is utilized to describe missing measurements by incorporating the measurement function $\psi_{k}$ with the following property-related statistical features: $p(\psi_{k}=1)$ $=E(\psi_{k})=p$ and $p\left(\psi_{k}=0\right)=E\left(\psi_{k}\right)=1-p$ [23]. When $\psi_{k}=1$ , the sensor obtains data with precision; conversely, it simply captures noise when $\psi_{k}=0,$ and no measurements are taken. Note that when referring to models (1) and (2) as reflective of the existence of missing measurements, the system receives data from the sensor at all times, and it is impossible to determine whether the data $\theta_{k}$ are obtained when $\psi_{k}=1$ or $\psi_{k}=0$ . Despite the fact that the nonlinear system with missing measurements has become increasingly prevalent in real-life situations owing to multiplicative noise $\psi_{k}$ , it complicates the attainment of optimal filtering outcomes.

This study aimed to utilize the concept of the least mean square error to construct an RCKF for nonlinear discrete systems represented by (1) and (2). The RCKF method is dependent on the robust M-estimation technique.

Bayesian filtering seeks to estimate the probability density function (PDF) of state variable $\lambda_{k}$ based on the sequence of all available measurements $\Theta_{k-1}=\left\{\theta_{1},\theta_{2},\cdots ,\theta_{k-1}\right\}$ up to time *k*. Thus, it is required to construct the posterior PDF $p(\lambda_{k}|\Theta_{k})$ and the prior PDF $p(\lambda_{k}|\Theta_{k-1})$ of the state variables $\lambda_{k}$ . That is, the condition PDF of $\lambda_{k}$ given $\Theta_{k}$ and $\Theta_{k-1}$ can be recursively computed using the provided solutions.

(3) $$\begin{array}{c} p\left(\lambda_{k}|\Theta_{k-1}\right)=\int p\left(\lambda_{k}|\lambda_{k-1}\right)p\left(\lambda_{k-1}|\Theta_{k-1}\right)d\lambda_{k-1}, \end{array}$$

(4) $$\begin{array}{c} p\left(\lambda_{k}|\Theta_{k}\right)=\frac{p\left(\theta_{k}|\lambda_{k}\right)p\left(\lambda_{k}|\Theta_{k-1}\right)}{p(\theta_{k}|\Theta_{k-1})}, \end{array}$$

**Figure 1:**
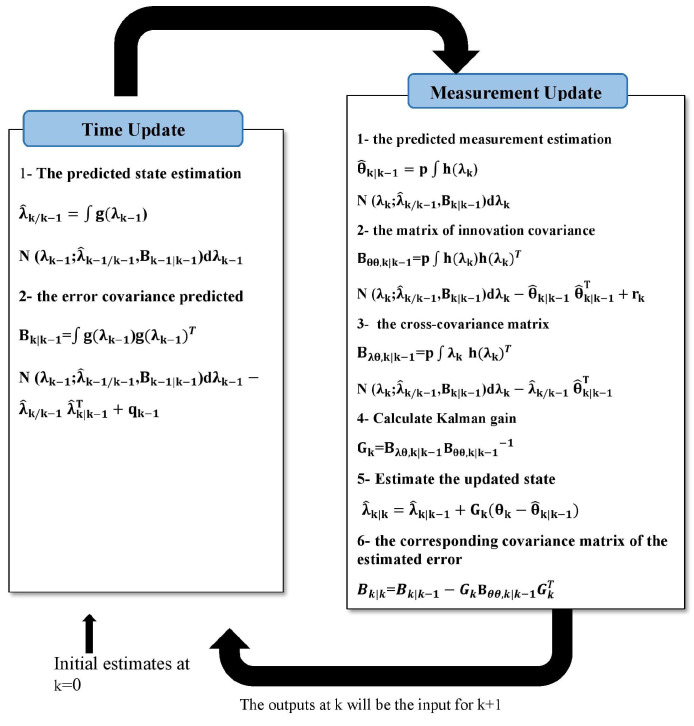
The algorithm of the KF.

Assuming that $p\left(\lambda_{k-1|k-1}|\Theta \right)\approx N\left({\hat{\lambda }}_{k-1|k-1},B_{k-1|k-1}\right)$ and $p\left(\lambda_{k|k-1}|\Theta \right)\approx N\left({\hat{\lambda }}_{k|k-1},B_{k|k-1}\right)$ , we can obtain the conditional probability densities in (3) and (4) by calculating the mean and covariance using the Kalman filter (KF) [24]. The KF has two stages of operation: time and measurement updates. While some sources use the terms “forecast” and “analysis”, others use “prediction” and “update” to describe these two stages. For details, see [25]; Figure 1 summarizes the algorithm of the KF.

## 3. Cubature Kalman Filter with Missing Measurements

Since the calculation of multivariate integrals is difficult, utilizing approximate methods is essential [16]. The CKF, invented by Arasaratnam et al. in 2009, is a Bayesian filter that provides an approximation for nonlinear filtering problems at the discrete time scale. It assumes that the predictive density of the combined state measurement follows a Gaussian distribution. The CKF employs the third-degree spherical-radial cubature rule to numerically compute integrals, scaling points linearly with the state vector dimension [15]. It effectively addresses complex nonlinear problems with high dimensions [26].

The classical form of the CKF is introduced below, comprising two distinct components—the measurement update and the time prediction [27]:

1. The time prediction is as follows:
  I The posterior probability distribution of a given k−1 time is
(5) $$p\left(\lambda_{k-1}|\Theta_{k-1}\right)=N\left(\lambda_{k-1}:{\hat{\lambda }}_{k-1|k-1},{\hat{B}}_{k-1|k-1}\right).$$
    By Cholesky decomposition, The expression denoting the error covariance at time k−1 , denoted as $B_{k-1|k-1}$ , is given by
(6) $$B_{k-1|k-1}=A_{k-1|k-1}A_{k-1|k-1}^{T},$$
    where $A_{k-1|k-1}$ denotes a diagonal time k−1 matrix.
  II Calculating the cubature points.
(7) $$lambda_{t,k-1|k-1}=A_{k-1|k-1}\zeta_{t}+{\hat{\lambda }}_{k-1|k-1},$$
    where $\lambda_{t,k-1|k-1}$ (t=1,2,⋯,2n) represents the system state of the *t*-th cubature point at time k−1 . The cubature points set is denoted as $\left[\zeta_{t}\right]$ and can be defined as
(8) $$\zeta_{t}=\sqrt{\frac{2n}{2}}{\langle 1\rangle }_{t}\;\;\;\;t=1,2,\cdots ,2n,$$
    where 2n is the number of cubature points or twice the state dimension; 〈1〉 refers to a set of problems; and ${\langle 1\rangle }_{t}$ is the *t*-th point in 〈1〉 .
  III Predicting state.
    The *t*-th cubature point’s predicted state from time k−1 to time *k* is defined as
(9) $$\lambda_{t,k|k-1}^{*}=g\left(\lambda_{t,k-1|k-1}\right).$$
    Then, the predicted state from time k−1 to time *k* is obtained from (9),
(10) $${\hat{\lambda }}_{k|k-1}=\frac{1}{2n}\sum_{t=1}^{2n}\lambda_{t,k|k-1}^{*},$$
    and its covariance is
(11) $$\begin{array}{cc} B_{k|k-1}= & \frac{1}{2n}\sum_{t=1}^{2n}\lambda_{t,k|k-1}^{*}\lambda_{t,k|k-1}^{*T}-\\ & {\hat{\lambda }}_{k|k-1}{\hat{\lambda }}_{k|k-1}^{T}+q_{k-1}. \end{array}$$
2. The measurement update is as follows, including the error covariance $B_{k|k-1}$ at time *k*:
  I Factorizing the CM of the error $B_{k|k-1}$ .
(12) $$B_{k|k-1}=A_{k/k-1}A_{k|k-1}^{T}.$$
  II Calculating the cubature points.
(13) $$\lambda_{t,k|k-1}=A_{k|k-1}\zeta_{t}+{\hat{\lambda }}_{k|k-1}.$$
  III Updating observation.
    The estimated observation of the *t*-th cubature point between epochs k−1 and *k* is denoted by
(14) $$\theta_{t,k|k-1}^{*}=h\left(\lambda_{t,k|k-1}\right).$$
    From (24), we can obtain the predicted observation of the *t*-th cubature point from time k−1 to k :
(15) $${\hat{\theta }}_{k|k-1}=\frac{p}{2n}\sum_{t=1}^{2n}\theta_{k|k-1}^{*},$$
    and its covariance and cross-covariance matrices are
(16) $$\begin{array}{cc} B_{\theta \theta ,k|k-1}= & \frac{p}{2n}\sum_{t=1}^{2n}\theta_{t,k|k-1}^{*}\theta_{t,k|k-1}^{*T}-\\ & {\hat{\theta }}_{k|k-1}{\hat{\theta }}_{k|k-1}^{T}+r_{k}, \end{array}$$
    and
(17) $$\begin{array}{cc} B_{\lambda \theta ,k|k-1}= & \frac{p}{2n}\sum_{t=1}^{2n}\lambda_{t,k|k-1}^{*}\theta_{t,k|k-1}^{*T}-\\ & {\hat{\lambda }}_{k|k-1}{\hat{\theta }}_{k|k-1}^{T}. \end{array}$$
  IV Calculating the Kalman gain.
(18) $$G_{k}=B_{\lambda \theta ,k|k-1}B_{\theta \theta ,k|k-1}^{-1}.$$
  V State update.
(19) $${\hat{\lambda }}_{k|k}={\hat{\lambda }}_{k|k-1}+G_{k}\left(\theta_{k}-{\hat{\theta }}_{k|k-1}\right).$$
  VI CM of the estimate error update.
(20) $$B_{k|k}=B_{k|k-1}+G_{k}B_{\theta \theta ,k|k-1}G_{k}^{T}.$$

**Figure 2:**
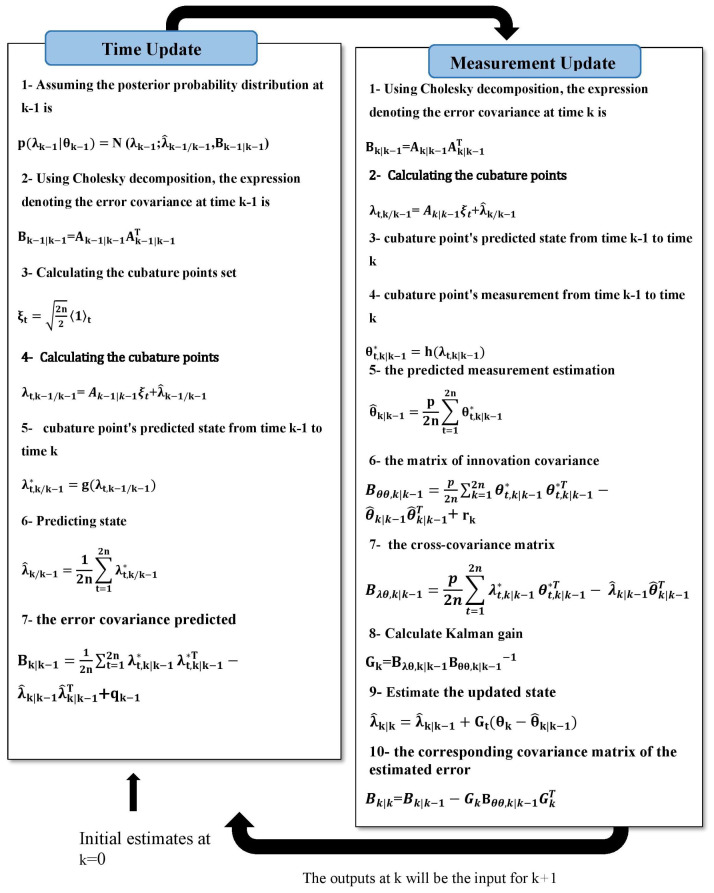
The algorithm of the CKF with missing measurements.

Figure 2 shows a comprehensive overview of the algorithm of the CKF with missing measurements.

## 4. Robust Cubature Kalman Filter with Missing Measurements

In the context of applying the CKF to a nonlinear system, it is imperative to possess a comprehensive understanding of the noise statistics associated with the device as well as its mathematical model. However, in the event that a filter is established based on an inaccurate mathematical model and noise statistics, there is a possibility of encountering a significant inaccuracy in the estimation of the system’s state or even the divergence of the estimation [16]. The robust M-estimation theory is a valuable technique for estimating unknown noise statistics [28]. Robust M-estimation can be employed to identify anomalies in state estimation. Additionally, the continuous updating of the statistical features of measurement noise enables the CKF to adapt to variations in the statistical characteristics of measurement noise in real time. The RCKF technique is formed by integrating Huber’s M-estimation theory with the conventional CKF model [26]. In this paper, this technique is used to deal with nonlinear systems with missing measurements. The algorithm will be derived in the subsequent sections. In contrast to the conventional CKF method, the RCKF technique selectively modifies and updates the appropriate representations within the measurement updating formula:

(21) $${\tilde{B}}_{\theta \theta ,k|k-1}=\frac{p}{2n}\sum_{t=1}^{2n}\theta_{t,k|k-1}^{*}\theta_{t,k|k-1}^{*T}-{\hat{\theta }}_{k|k-1}{\hat{\theta }}_{k|k-1}+{\tilde{r}}_{k},$$

where ${\tilde{B}}_{\theta \theta ,k|k-1}$ can be obtained by estimating a weight matrix *B* using an absence of difference M-estimation approach, and ${\tilde{r}}_{k}$ is equal to the measurement noise variance matrix $r_{k}$ . That is,

(22) $${\tilde{r}}_{k}={\tilde{B}}^{-1},$$

where the matrix $\tilde{B}$ is created using Huber’s approach [29]. This process depends on considering the KF as a linear regression problem, as explained in [28], that can be solved with resistance and robust efficiency using the M-estimation. This minimizes the cost function as follows:

(23) $$C\left(\lambda_{k}\right)=\sum_{t=1}^{2n}\rho \left(b_{t}^{\prime }\right).$$

Here, $b_{t}^{\prime }$ denotes the residue vector’s *t*-th component

(24) $$b_{t}^{\prime }=b_{t}/A_{bt},$$

where $b_{t}$ is a residual component associated with the observation quantity $\theta_{k}$ , and $A_{bt}$ is the mean square error associated with $b_{t}$ . The expressions $A_{bt}$ and $b_{t}$ are used in practice because the covariance matrix of the measurement residuals is acquired from (16), which is the variable quantity $B_{\theta \theta ,k|k-1}$ previous to being adjusted:

(25) $$A_{bt}={\left(B_{\theta \theta ,k|k-1}\right)}_{tt},$$

(26) $$b_{t}={\left(\theta_{k}-{\hat{\theta }}_{k|k-1}\right)}_{t}.$$

The score function $\rho (b_{t}^{\prime })$ is defined as follows [30,31]:

(27) $$\begin{array}{c} \;\;\;\;\;\;\;\;\;\;\;\;\;\;\;\;\;\;\;\rho \left(b_{t}^{\prime }\right)=\left\{\begin{array}{cc} {b_{t}^{\prime }}^{2} & \;\;\;\;\;\mathrm{if}\;|b_{t}^{\prime }|\leq c\\ c|b_{k}^{\prime }|-\frac{1}{2}c^{2} & \;\;\;\;\mathrm{otherwise}, \end{array}\right. \end{array}$$

where *c* is a constant that is typically between 1.3 and 2.0 [16]. When the partial derivative of (24) is set to zero,

(28) $$\sum_{t=1}^{2n}\frac{\partial \rho (b_{t}^{\prime })}{\partial (b_{t}^{\prime })}.\frac{\partial (b_{t}^{\prime })}{\lambda_{t,k}}\;\;\;\;\;k=1,2,\cdots ,n,$$

where $\lambda_{t,k}$ is the state vector in the *t*-th component. Following

(29) $$w_{t}=\frac{\partial (b_{t}^{\prime })}{b_{t}^{\prime }\partial b_{t}^{\prime }},$$

we can obtain the formula

(30) $$\begin{array}{c} w_{t}=\left\{\begin{array}{cc} 1 & \;\;\;\;\;\;\;\;\;\;\;\;\mathrm{if}\;|b_{t}^{\prime }|\leq c,\\ \frac{c}{|b_{t}^{\prime }|} & \;\;\;\;\;\;\;\;\;\;\;\;\;\mathrm{otherwise}. \end{array}\right. \end{array}$$

**Figure 3:**
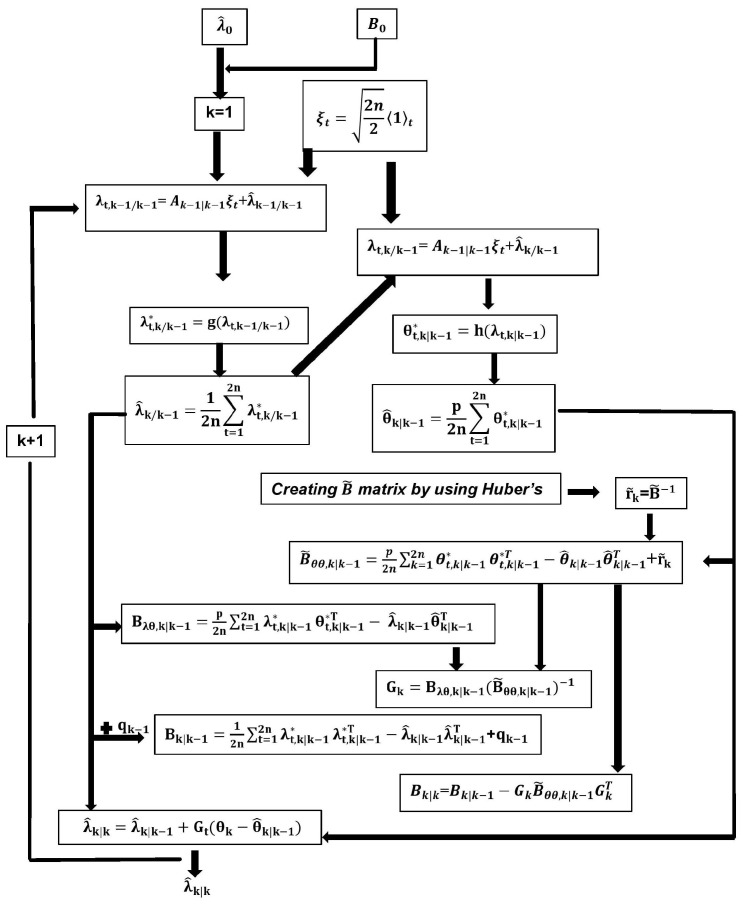
The algorithm of the RCKF with missing measurements.

Depending on (30), the Huber approach will determine which diagonal components of $\tilde{B}$ are positive. An identical expression is provided below:

(31) $$\begin{array}{c} {\tilde{B}}_{t,t}=\left\{\begin{array}{cc} \frac{1}{A_{t,t}} & \;\;\;\;\;\;\;\;\;\;\;\;\mathrm{if}\;|\frac{b_{t}}{A_{bt}}|=|b_{t}^{\prime }|\leq c\\ \frac{c}{A_{t,t}|b_{t}^{\prime }|} & \;\;\;\;\;\;\;\;\;\;\;\;\;\mathrm{otherwise}, \end{array}\right. \end{array}$$

(32) $$\begin{array}{c} {\tilde{B}}_{t,j}=\left\{\begin{array}{cc} \frac{1}{A_{t,j}} & \;\;\;\;\;\;\;\;\;\;\;\;\mathrm{if}\;|b_{t}^{\prime }|<c,|b_{j}^{\prime }|<c\\ \frac{c}{A_{t,j}\max\left(|b_{t}^{\prime }|,|b_{j}^{\prime }|\right)} & \;\;\;\;\;\;\;\;\;\;\;\;\;\mathrm{otherwise}, \end{array}\right. \end{array}$$

where the diagonal and off-diagonal elements in the matrix $\tilde{B}$ are denoted as ${\tilde{B}}_{t,t}$ and ${\tilde{B}}_{t,j}$ , respectively. Similarly, the diagonal and off-diagonal elements in the measurement noise $r_{k}$ are represented as $A_{t,t}$ and $A_{t,j}$ , respectively. The element $A_{t,j}$ is equal to zero due to the fact that the matrix representing the covariance of measurement noise is diagonal. The symbol $b_{t}$ represents the measurement residual, while $b_{t}^{\prime }$ denotes the standard residual error. Additionally, $A_{bt}$ represents the mean variance of $b_{t}$ . The algorithm for the given RCKF with missing measurements is depicted in Figure 3.

## 5. Metrics of Performance

When evaluating a new filter, it is commonly compared to standard filters using benchmark workloads. The root mean square error (RMSE) is a widely used metric for evaluation [32]. Still, it only assesses the filter’s output at the initial instance, specifically the conditional mean of the state [33]. In this study, we not only compared the state estimate ${\hat{\lambda }}_{k|k}$ but also the conditional mean of the estimated error. The non-credibility index (NCI) served as a credibility metric for comparing the filter’s efficiency in producing the conditional mean [34].

1. Root mean square error (RMSE).
  The RMSE for the state estimate ${\hat{\lambda }}_{k|k}$ generated utilizing M Monte Carlo simulations at time instant *k* is as follows:
(33) $$\;\;\;\;\;\;\;\;\;\;RMSE\left({\hat{\lambda }}_{k|k}\right)=\sqrt{\frac{1}{M}\sum_{t=1}^{M}{\left(\lambda_{k}\left(t\right)-{\hat{\lambda }}_{k|k}\right)}^{T}\left(\lambda_{k}\left(t\right)-{\hat{\lambda }}_{k|k}\right)},$$
  where the estimate of $\lambda_{k}\left(t\right)$ at the *t*-th Monte Carlo simulation is ${\hat{\lambda }}_{k|k}$ .
2. Non-credibility index.
  In order to calculate the NCI, we compared the estimator’s normalized squared estimation error, which is defined as
(34) $$\;\;\;\;\epsilon_{k|k}\left(t\right)={\left(\lambda_{k}\left(t\right)-{\hat{\lambda }}_{k|k}\right)}^{T}B_{k|k}{\left(t\right)}^{-1}\left(\lambda_{k}\left(t\right)-{\hat{\lambda }}_{k|k}\right),$$
  with the credible estimator’s normalized squared estimation error, expressed as [35]
(35) $$\;\;\;\;\epsilon_{k|k}^{*}\left(t\right)={\left(\lambda_{k}\left(t\right)-{\hat{\lambda }}_{k|k}\right)}^{T}\phi_{k|k}^{-1}\left(\lambda_{k}\left(t\right)-{\hat{\lambda }}_{k|k}\right),$$
  where $\phi_{k|k}$ is the mean square error (MSE) computed by $(\frac{1}{M}\sum_{t=1}^{M}{\left(\lambda_{k}\left(t\right)-{\hat{\lambda }}_{k|k}\right)}^{T}\left(\lambda_{k}\left(t\right)-{\hat{\lambda }}_{k|k}\right)$ . The NCI is described as
(36) $$NCI\left(k\right)=\frac{10}{M}\sum_{t=1}^{M}|\log_{10}\frac{\epsilon_{k|k}\left(t\right)}{\epsilon_{k|k}^{*}\left(t\right)}|.$$
  The NCI can measure the estimator’s credibility. That is, the estimator’s CM is close to the MSE $\left(\phi_{k|k}\right)$ . The lower the NCI score, the more reliable the estimator; therefore, an NCI score of zero indicates an entirely credible estimator.

## 6. Numerical Experiments

This section presents a comparative analysis of the performance of the EKF, UKF, CKF, and RCKF through the examination of two examples. The simulation of signal and observation values was conducted using MATLAB, and alternate filtering estimates will be presented. The determination of appropriate model parameters and how we conducted a comprehensive study to compare the methods are also explained.

### 6.1. Model Specifications

This part describes the benchmark models used to compare the methods. The consistently accelerating moving-target tracking model monitors a moving target with missing measurements and has many applications. The numerical representations were carried out using two different models: the UNGM [36] and BOT [37,38]. We applied the two models in two scenarios regarding the missing measurements: $p(\psi_{k}=1)=0.7$ and $p(\psi_{k}=1)=0.8$ .

1. The UNGM.
  This model is described as follows:
(37) $$\begin{array}{cc} \lambda_{k}= & 0.5\lambda_{k-1}+25\frac{\lambda_{k-1}}{1+\lambda_{k-1}^{2}}+8\cos\left(1.2\left(k-1\right)\right)\\ & +\delta_{k}, \end{array}$$
  and
(38) $$\theta_{k}=\psi_{k}\times \frac{\lambda_{k}^{2}}{20}+\xi_{k},$$
  where $\delta_{k}\;∼N\left(0,1\right)$ , $\xi_{k}\;\;∼N\left(0,1\right)$ , $\lambda_{0}\;∼N\left(0.1,1\right)$ , probability $p(\psi_{k}=1)=0.7$ , and $p(\psi_{k}=1)=0.8$ .
2. BOT.
  There are two states inside the bearing-only tracking (BOT) paradigm, with the state $\lambda_{k}={\left[\lambda_{1,k}\;\;\lambda_{2,k}\right]}^{T}$ displaying a tracked target’s positioning in Cartesian coordinates. Its nonlinear model is as follows:
(39) $$\lambda_{k}=\left[\begin{array}{cc} \;\;0.9 & 0\\ 0 & 1 \end{array}\right]\times \lambda_{k-1}+\delta_{k},$$
(40) $$\theta_{k}=\psi_{k}\left(\arctan\left(\frac{\lambda_{2,k}-\sin\left(k\right)}{\lambda_{1,k}-\cos\left(k\right)}\right)\right)+\xi_{k},$$
  where $\delta_{k}∼N\left(0,0.001\times \left[\begin{array}{cc} 1 & 0\\ 0 & 3 \end{array}\right]\right)$ , $\xi_{k}∼N\left(0,0.005\right)$ , $\lambda_{0}∼N\left({\left[20\;\;\;5\right]}^{T},0.1\times \left[\begin{array}{cc} 1 & 0\\ 0 & 3 \end{array}\right]\right)$ , and $p(\psi_{k}=1)=0.7$ , $p(\psi_{k}=1)=0.8$ .

### 6.2. Experiment and Analysis

**Figure 4:**
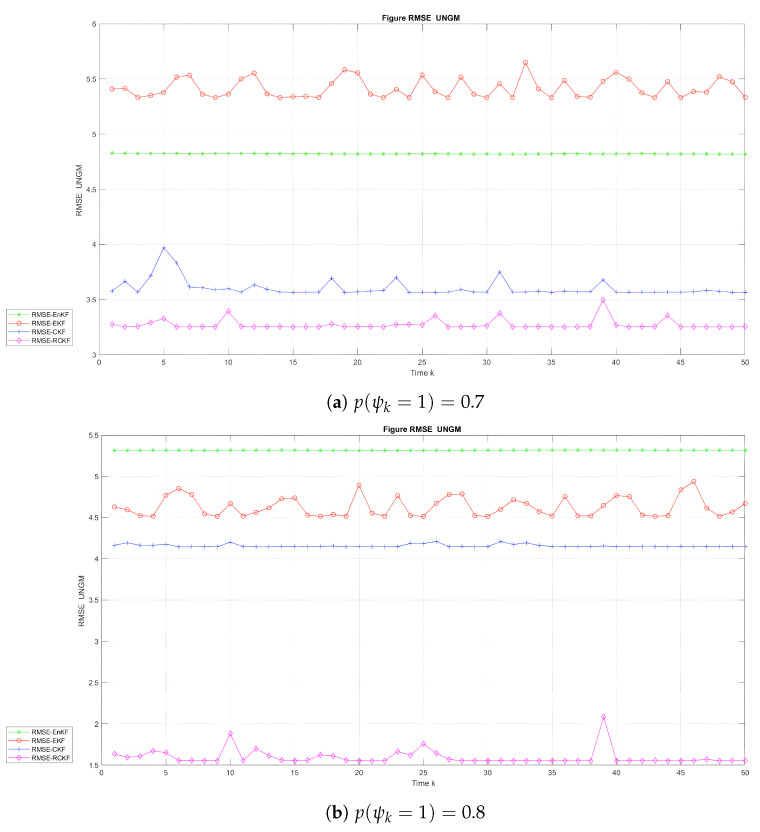
RMSE with time in the UNGM.

**Figure 5:**
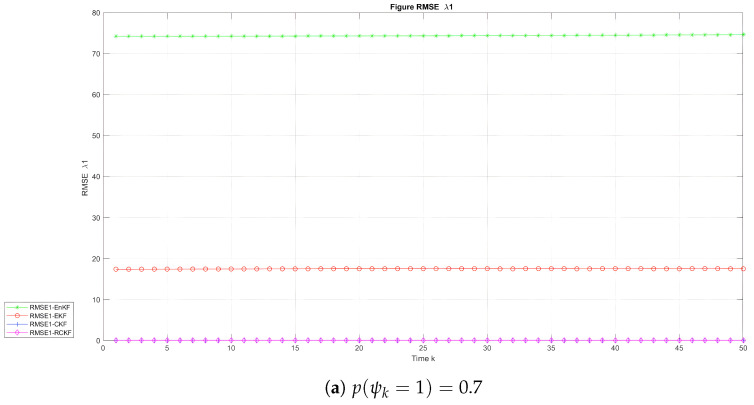

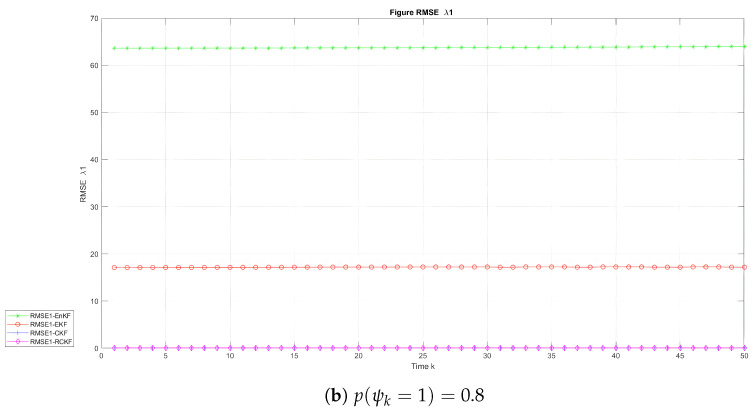
RMSE with time in the BOT model for $\lambda_{1}$ .

**Figure 6:**
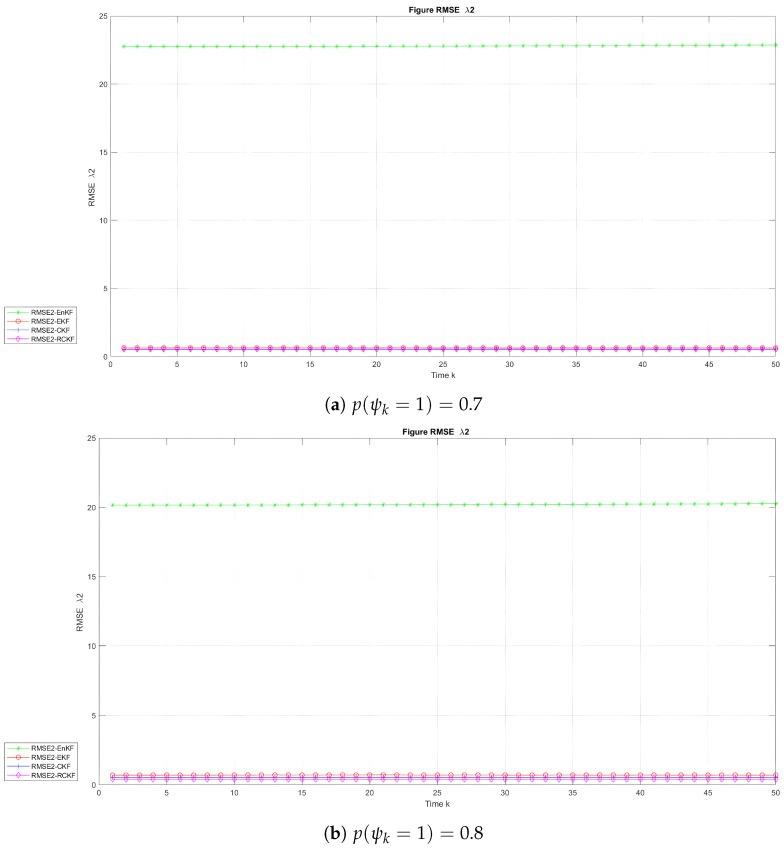
RMSE with time in the BOT model for $\lambda_{2}$ .

The UNGM and BOT examples underwent evaluation through 100 independent Monte Carlo simulations with 50 time intervals and two scenarios regarding the missing measurements: $p(\psi_{k}=1)=0.7$ and $p(\psi_{k}=1)=0.8$ . Figure 4, Figure 5 and Figure 6 depict the evolution of the RMSE with time for the UNGM, BOT $\lambda_{1}$ , and BOT $\lambda_{2}$ , respectively. Table 2 and Table 3 provide the average RMSE (ARMSE) for the UNGM and BOT. The RCKF exhibited the highest accuracy and achieved the lowest RMSE for the UNGM example in the two scenarios, as shown in Figure 4. The RCKF consistently outperformed the other methods in filtering precision. Also, Table 2 shows that the RCKF had the lowest ARMSE, with values of 3.27 and 1.60 when $p(\psi_{k}=1)=0.7$ and $p(\psi_{k}=1)=0.8$ , respectively. For BOT state $\lambda_{1}$ , both the RCKF and CKF showed a comparable estimating accuracy and outperformed the EKF and EnKF in the two scenarios. Figure 5 and Table 3 indicate that the RCKF and CKF had the same ARMSE in the two scenarios, while in the state $\lambda_{2}$ , the RCKF and CKF demonstrated similar estimation accuracies when $p(\psi_{k}=1)=0.7$ . The RCKF had the highest accuracy when $p(\psi_{k}=1)=0.8$ , achieving the lowest ARMSE of 0.40. Table 2 also reveals that the RCKF, CKF, and EKF exhibited similar ARMSE values.

**Figure 7:**
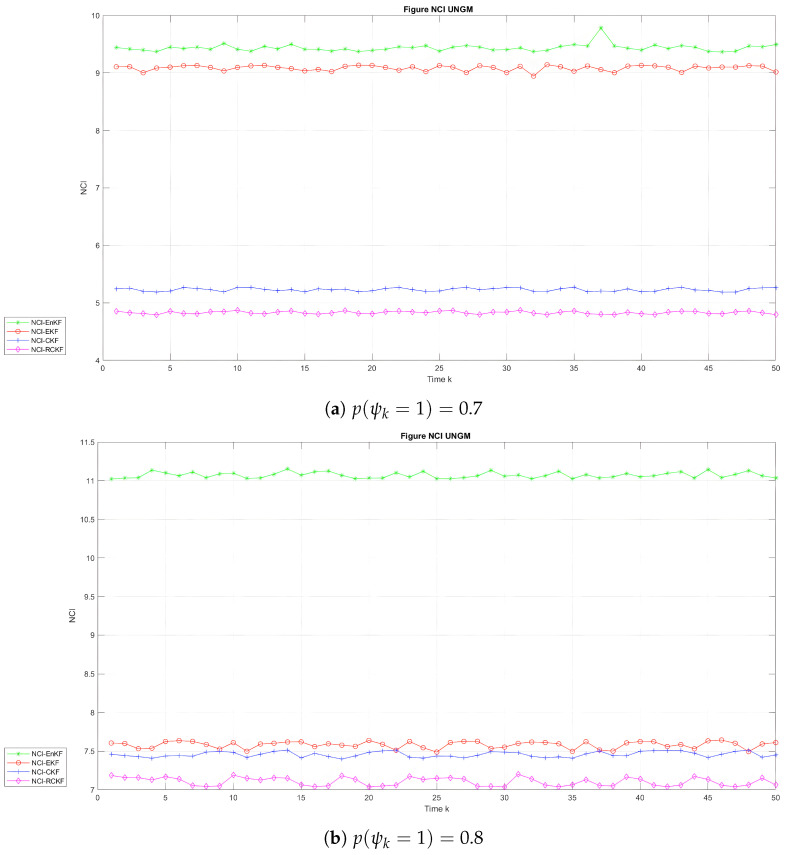
NCI with time in the UNGM.

**Figure 8:**
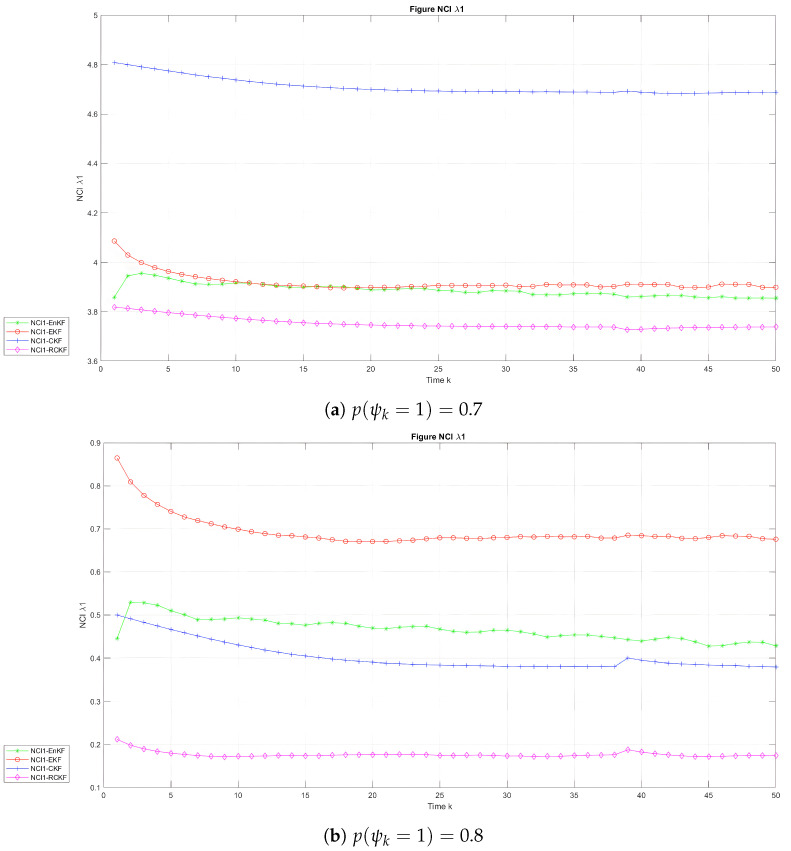
NCI with time in the BOT model for $\lambda_{1}$ .

**Figure 9:**
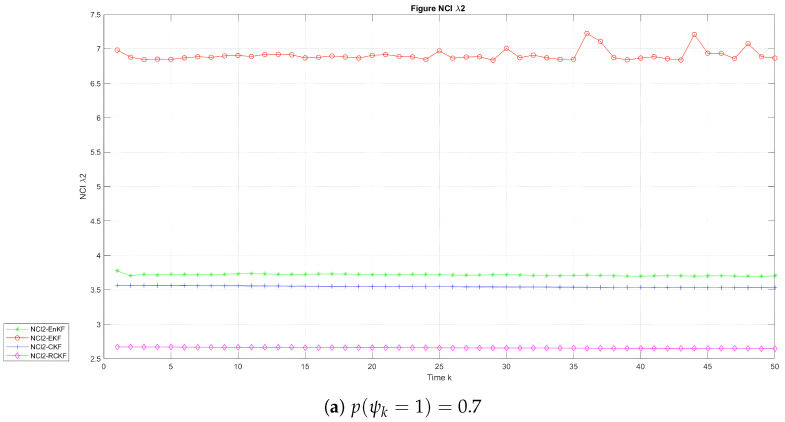

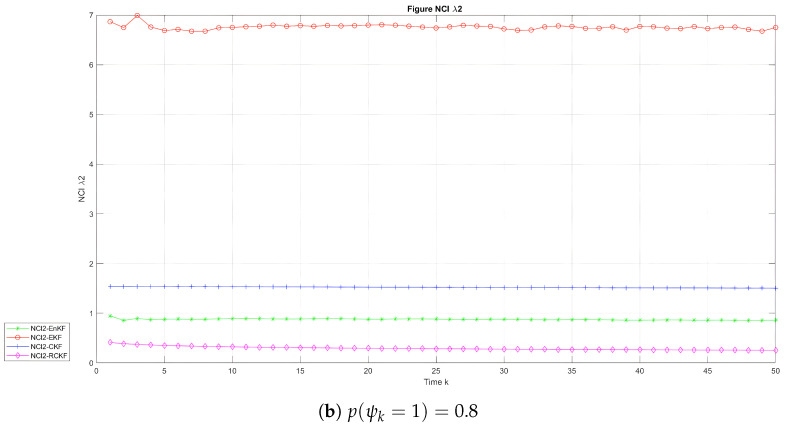
NCI with time in the BOT model for $\lambda_{2}$ .

Figure 7 depicts the temporal progression of the NCI in the two scenarios for the UNGM, while Figure 8 and Figure 9 exhibit the corresponding evolution for BOT states $\lambda_{1}$ and $\lambda_{2}$ in the two scenarios, respectively. Table 4 and Table 5 present the average NCI (ANCI) for the UNGM and BOT in both states. According to the findings in Figure 7, the RCKF exhibited superior outcome credibility for the UNGM in the two scenarios, as seen by the lowest scores. According to Table 4, the RCKF approach demonstrated outstanding performance compared to the other methods regarding the average filtering credibility, as indicated by the ANCI scores of 4.83 and 7.10 when $p(\psi_{k}=1)=0.7$ and $p(\psi_{k}=1)=0.8$ , respectively. Similarly, it can be observed from Figure 8 and Figure 9 that the RCKF demonstrated the highest level of reliability in the context of the BOT example. Additionally, it is worth noting that the NCI score was found to be the lowest in both states. According to Table 5, the relative credibility Kalman filter (RCKF) demonstrated exceptional performance in terms of average filtering credibility in both states. Specifically, the ANCI values for $\lambda_{1}$ and $\lambda_{2}$ were reported as 3.75 and 2.65, respectively, when $p(\psi_{k}=1)=0.7$ , while the ANCI values were 0.18 and 0.29 when $p(\psi_{k}=1)=0.8$ , indicating the superiority of the RCKF method compared to other approaches in the two scenarios.

## 7. Conclusions

This study presented the RCKF as a filter for nonlinear systems with missing measurements. In order to accomplish this objective, we combined Huber’s M-estimation theory with the conventional CKF for nonlinear systems with missing observations and developed the filter using a recursive method. We demonstrated the effectiveness of the proposed method through two examples, the UNGM and BOT, and compared it with the EKF, EnKF, and CKF. The results showed that the RCKF provided more precise and credible outcomes compared to the other methods, with the highest accuracy observed in the UNGM example. Also, in the BOT example, the RCKF exhibited essentially superior accuracy to other methods. In general, compared to traditional techniques such as the EKF, EnKF, and CKF, the RCKF demonstrated the best accuracy and credibility for nonlinear systems with missing measurements.

Future research will focus on extending the RCKF to capture missing measurement phenomena through a general Markov chain rather than a Bernoulli sequence of identical independent distributions. Additionally, we propose using the RCKF as an alternate approach for estimating the state of nonlinear systems when the system noises follow a non-Gaussian distribution instead of a Gaussian distribution. 

## Figures and Tables

**Table 1:** Comparison of relevant works.

Authors	Method	Results
Tiancheng Li et al. [17]	Huber’s M-estimation-based robust CKF and robust square root CKF adapted to anomalous measurement noise using innovation covariance comparison.	Simulations demonstrated the superior performance in terms of accuracy, robustness, and reliability compared to standard methods for target tracking.
Zhao et al. [16]	Robust adaptive CKF to reduce kinematic model errors through covariance adjustment and dynamic disturbance processing.	The experiment confirmed the proposed strategy’s effectiveness in dynamic systems with high dynamics and weak signals.
Cui Bingbo et al. [18]	RCKF enhanced GNSS/INS accuracy in GNSS-denied environments by considering noise using missing observations.	Numerical experiments and field tests demonstrated the RCKF’s superior robustness compared to the CKF and EKF.
Xiangzhou Ye et al. [19]	Adaptive robust CKF (ARCKF) based on the H-infinity CKF by incorporating two adaptable algorithm components to address erroneous system models and noise statistics.	Simulations favored the recommended approach over the HCKF for handling model errors and aberrant observations.

**Table 2:** The UNGM average RMSE.

Method	Average RMSE When $p(\psi_{k}=1)=0.7$	Average RMSE When $p(\psi_{k}=1)=0.8$
EKF	5.41	4.63
EnKF	4.82	5.32
CKF	3.60	4.16
RCKF	3.27	1.60

**Table 3:** BOT average RMSE.

Method	Average RMSE $\lambda_{1}$ When $p(\psi_{k}=1)=0.7$	Average RMSE $\lambda_{1}$ When $p(\psi_{k}=1)=0.8$	Average RMSE $\lambda_{2}$	Average RMSE $\lambda_{2}$ When $p(\psi_{k}=1)=0.8$
EKF	17.50	17.19	0.64	0.71
EnKF	74.38	63.78	22.79	20.20
CKF	0.067	0.07	0.55	0.54
RCKF	0.057	0.07	0.53	0.40

**Table 4:** The UNGM average NCI.

Method	Average NCI When $p(\psi_{k}=1)=0.7$	Average NCI When $p(\psi_{k}=1)=0.8$
EKF	9.08	7.58
EnKF	9.44	11.07
CKF	5.23	7.46
RCKF	4.83	7.10

**Table 5:** BOT average NCI.

Method	Average NCI $\lambda_{1}$ When $p(\psi_{k}=1)=0.7$	Average NCI $\lambda_{1}$ When $p(\psi_{k}=1)=0.8$	Average NCI $\lambda_{2}$ When $p(\psi_{k}=1)=0.7$	Average NCI $\lambda_{2}$ When $p(\psi_{k}=1)=0.8$
EKF	3.91	0.69	6.91	6.76
EnKF	3.89	0.47	3.72	0.88
CKF	4.71	0.40	3.53	1.52
RCKF	3.75	0.18	2.65	0.29

## Data Availability

Data are contained within the article.

## Abbreviations

The following abbreviations are used in this manuscript:

**Abbreviations**		
RCKF	Robust cubature Kalman filter	
CKF	Cubature Kalman filter	
EKF	Extended Kalman filter	
UKF	Unscented Kalman filter	
EnKF	Ensemble Kalman filter	
CM	Covariance matrix	
MSE	Mean square error	
RMSE	Root mean square error	
NCI	Non-credibility index	
UNGM	Univariate Non-stationary Growth Model	
BOT	Bearing-only tracking	
**Symbols**		
$\lambda_{k}$	The state vector	n×1
$\theta_{k}$	The measurement vector	m×1
g(.)	Nonlinear function of the state	n×n
h(.)	Nonlinear function of the measurement	m×m
$\delta_{k-1}$	The process noise	n×1
$\xi_{k}$	The measurement noise	m×1
$q_{k-1}$	The covariance matrix of $\delta_{k-1}$	n×n
$r_{k}$	The covariance matrix of $\xi_{k}$	m×m
$\psi_{k}$	Factor of missing measurement	1×1
${\hat{\lambda }}_{k|k-1}$	The predicted state estimation	n×1
${\hat{\theta }}_{k|k-1}$	The predicted measurement estimation	m×1
$B_{k|k-1}$	Predicted error covariance estimation	n×n
$B_{\theta \theta ,k|k-1}$	Estimated matrix of innovation covariance	m×m
$B_{\lambda \theta ,k|k-1}$	Estimated cross-covariance matrix	n×m
$G_{k}$	Kalman gain	n×m
${\hat{\lambda }}_{k|k}$	Estimated update state	n×1
$\zeta_{t}$	The cubature point	n×1
${\tilde{B}}_{\theta \theta ,k|k-1}$	Estimated matrix of innovation covariance using an absence of difference M-estimation approach $\xi_{k}$	m×m
$b_{t}$	Residue vectors *t*-th	m×1
$b_{t}^{\prime }$	Residue vectors *t*-th	m×1
$A_{bt}$	Mean variance of $b_{t}$	m×m
